# Mitigating indoor risk of airborne infections using CO_2_ and PM measurements in university classroom: the MIRAI project

**DOI:** 10.1007/s40201-026-00984-2

**Published:** 2026-05-30

**Authors:** Riccardo Mazzoli, Tommaso Filippini, Grazia Ghermandi, Marco Vinceti, Alessandro Bigi

**Affiliations:** 1 Environmental, Genetic and Nutritional Epidemiology Research Center (CREAGEN), Department of Biomedical, Metabolic and Neural Sciences, Section of Public Health, University of Modena and Reggio Emilia, Modena, Italy; 2 School of Public Health, University of California Berkeley, Berkeley, CA USA; 3 DIEF - Department of Engineering ‘Enzo Ferrari’, University of Modena and Reggio Emilia, Modena, Italy; 4 Department of Epidemiology, Boston University School of Public Health, Boston, MA USA; 5 Department of Biomedical, Metabolic and Neural Sciences, University of Modena and Reggio Emilia, 287 Via Campi, 41125 Modena, Italy

**Keywords:** airborne infection, carbon dioxide, indoor air quality, infectious disease, particulate matter

## Abstract

**Background:**

Indoor air quality (IAQ) impacts well-being and the spread of airborne diseases, particularly in multi-occupancy environments like classrooms. Carbon dioxide (CO_2_) and particulate matter (PM) levels have been used in continuous monitoring as indicators of indoor air quality.

**Methods:**

We used cost-effective sensors to assess levels of CO2 and PM in two Italian university classrooms (Unit 1 and Unit 2), differing in size and ventilation, from March 2022 to May 2023. For CO2 and PM monitoring we used respectively ARANET4 sensors and Optical Particle Counters (OPC) assessing aerosol diameter size distribution between 350 nm and 40 μm in order to evaluate trends in IAQ and the influence of environmental factors including ventilation, occupancy, and season.

**Results:**

The naturally ventilated classroom (Unit 1) exhibited higher CO2 and PM concentrations with greater variability, whereas the HVAC-supported one (Unit 2) maintained more consistent air quality but faced occasional spikes. Occupancy also affected CO2 and PM levels, with higher variability in Unit 1 characterized by lower size and generally full occupancy. Seasonal trends highlighted increased PM levels during colder months due to reduced ventilation in both units.

**Conclusions:**

In this feasibility study, variations in building design, ventilation strategies, and occupancy dynamics were associated with corresponding patterns in IAQ, supporting the use of low-cost, user-friendly sensors as practical tools to characterize ventilation performance and to inform interventions aimed at safer learning spaces. Implementation of CO2 and PM measurements as proxy indicators of ventilation adequacy and of potential airborne infection risk, along with outreach programs and guidelines to educate teachers and students about relevance of IAQ assessment is recommended to promote occupant health and mitigate risk of airborne disease transmission.

**Supplementary Information:**

The online version contains supplementary material available at 10.1007/s40201-026-00984-2.

## Introduction

Maintaining good Indoor Air Quality (IAQ) is essential for well-being in daily living places, as people spend a large portion of their time inside [1, 2]. Poor indoor air quality can lead to a range of issues, from minor discomforts such as headaches and fatigue, to more serious health concerns, such as allergy flare ups and respiratory diseases [3–7].

The risk of infection from airborne pathogens is especially high in multi-occupancy indoor environments, such as office, classroom, and public transportation, where many individuals share the same air space for extended periods [8–10]. Both personal (e.g., viral load, individual susceptibility, modality of exposure) and environmental (e.g., airflow patterns, temperature, humidity) factors contribute to modify infection risk [11]. While personal factors are inherently challenging to measure with cost-effective methods continuously and in real-time, environmental factors provide a more practical approach for continuous monitoring and assessment of infection risk, evaluating the effectiveness of mechanical or natural ventilation [12, 13].

Carbon dioxide (CO_2_) and particulate matter (PM) levels have been used as indicators of air quality, with recent studies highlighting the value of continuous monitoring to identify spaces with inadequate ventilation — especially those where people congregate for extended periods, such as office and classroom [14, 15]. Furthermore, elevated PM concentrations are linked to increased mortality from stroke, acute respiratory infections and heart disease [16–18]. After detecting these poorly ventilated areas, steps can be taken to improve air circulation, thus enhancing overall indoor air quality and reducing the risk of exposure to airborne contaminants, including infectious agents.

The spread of COVID-19, which is mainly transmitted through droplets and aerosols, has been greatly facilitated within indoor spaces, which have been identified as the main settings for infection [19, 20]. Not surprisingly, improved indoor air quality (IAQ), achieved through better ventilation, has been linked to a reduced risk of COVID-19 infection [21–24]. However, although the COVID-19 pandemic prompted and renewed the attention to ventilation as a public health intervention, the relevance of indoor air quality to airborne respiratory infection extends well beyond SARS-CoV-2. Other common seasonal respiratory pathogens, including rhinovirus, influenza viruses and respiratory syncytial virus (RSV), are also transmitted in shared indoor environments, and their circulation imposes a substantial annual burden of morbidity, hospitalization and absenteeism in schools and workplaces [25–28].

The advent of portable, accurate, low-cost sensors has advanced considerably the capacity to monitor air quality in real-time. The use of these sensors can be particularly impactful especially in multi-occupancy spaces, such as classrooms and offices, where the risk of airborne disease transmission is higher. These sensors allow for the immediate detection of areas of poor ventilation or where air quality may be compromised, prompting timely corrective interventions (such as increasing ventilation or adjusting air conditioning systems) [29, 30].

For these reasons, we conducted a feasibility study aimed at evaluating the year-round, real-world implementation of a low-cost sensor system for both CO_2_ and PM in university classrooms. The study is intended to characterize the feasibility, the operational challenges and the type of information that a low-cost monitoring strategy can provide.

## Methods

### Experimental setting

In this study, we employed two types of sensors. To evaluate CO_2_ levels, we used the ARANET4 sensor (SAF Tehnika JSC, Riga, Latvia), a wireless indoor low-cost CO_2_ monitor using Non-Dispersive InfraRed (NDIR) method, a state-of-art technique for detecting gas concentrations [31]. These devices are factory-calibrated and according to the manufacturer, single CO_2_ readings have an accuracy of ± 30 ppm ± 3% of the reading, and were proved to have a satisfactory performance from a laboratory intercomparison study [32].

We measured air PM using two Optical Particle Counters (OPC) OPC-N3 (Alphasense-AMETEK, Cambridge, UK). These devices use a fan to collect the air (total flow 5.5 L/min and a sample flow 0.2 L/min), while a diode laser illuminates individual aerosol particles and a photodiode records the intensity of the scattered light [33]. The intensity of scattering is used to derive the size of the particle, while the pulse of light is used to count the particles. OPC-N3 provides the aerosol size distribution between approximately 350 nm and 40 μm, divided in 24 bins [34], and this information is used to calculate the particulate mass concentration (PM), generally assuming a spherical particle shape and standard aerosol density and refractive index.

### Data measurement

We installed these sensors within two classrooms of the University of Modena and Reggio Emilia, specifically in the ‘Modena Campus’, one at the Section of Public Health of the Department of Biomedical, Metabolic and Neural Sciences (Unit 1: 4°37’52.0"N 10°56’34.5"E) and one at the Engineering Department (Unit 2: 44°37’47.0"N 10°56’55.1"E). Characteristics of classrooms (location, size, heating system) are reported in the Table 1. In particular, main differences are that Unit 1 has a capacity of 25 students using a wall radiator heating system, while Unit 2 can accommodate up to 228 students and has a central Heating, Ventilation and Air Conditioning (HVAC) system.

**Table 1:** Characteristics of classrooms

Location	Unit 1	Unit 2
	Public health department	Engineering department
Maximum No. of students	25	228
Size (m^2^)	42.78	235.1
Volume (m^3^)	141.17	919.24
Heating system	Wall Radiator	HVAC
Ventilation	Window	HVAC
Number of doors and windows	1 door, 3 windows	4 doors, 5 windows
Orientation	South-West	North-West

The data measurement campaign started soon after the end of COVID-19 state of emergency and the re-opening of school system at full capacity, from March 2022 to May 2023. ARANET4 sensors were placed in Unit 1 from March 18, 2022, to January 13, 2023, and in Unit 2 from October 21, 2022, to July 4, 2023. OPC-N3 devices were active in Unit 1 from April 11, 2022, to March 15, 2023, and in Unit 2 from May 4, 2022, to May 3, 2023. The sensors were mounted on the wall in a location to ensure a comprehensive coverage of the IAQ of the room and an accurate monitoring of CO_2_ and PM concentrations throughout the academic semesters. Additionally, indoor air temperature (°C) and relative humidity (RH%) were measured through the Aranet4 unit.

For CO_2_, temperature and relative humidity (RH%) data collection, we used the Aranet4 App, an application specifically developed to collect and visualize data within a timeframe of 14 days.

OPCs were connected to a RaspberryPi microcomputer and both were protected inside a small box, with a proper inlet for air sampling. The data were collected at 2 s time resolution, averaged to 1 min, transmitted to a central server. The cover of the box also had a QR code pointing to the project webpage (see paragraph on communication programme).

In each unit, the ARANET4 CO_2_ sensor and the OPC-N3 PM unit were installed at approximately 1.5 m above floor level, on the wall opposite the windows and away from the door, at a distance of at least 2 m from the nearest occupant in both units. This placement followed standard and sensor-specific recommendations for indoor air monitoring, and was intended to capture air representative of the occupants’ breathing zone while limiting short-circuit effects from doors, windows, and air-supply diffusers. The number of monitoring points per room was constrained by the availability of OPC-N3 units specifically configured for the project. Real-time occupancy counts were not collected; instead, the fixed teaching schedules of the two classrooms were used as a structural proxy for occupancy.

Throughout the manuscript CO_2_ is reported in ppm, as it is the unit used in guidelines and directly reported by the sensor, while particulate matter is reported in µg/m^3^, consistent with regulatory standards. For reference, in air at 25.0 °C and 1 atm, 1 ppm CO_2_ corresponds to approximately 1.80 mg/m^3^.

### Data analysis

The collected CO_2_ and aerosol data was analyzed to identify trends and patterns in indoor air quality, to generate reports detailing the frequency and duration of CO_2_ levels exceeding the established thresholds. We categorized collected data into “weekdays,” referring to days with scheduled classes, and “weekends,” which included weekends, public holidays, and departmental closure periods. We used R (R version 4.4.1, R Foundation for Statistical Computing, Vienna, Austria, 2024) and R Studio (R Studio version 2024.04.1 + 748, Posit Software PBC, Boston, 2024) for all data analysis and presentation.

### Communication program

**Fig. 1:**
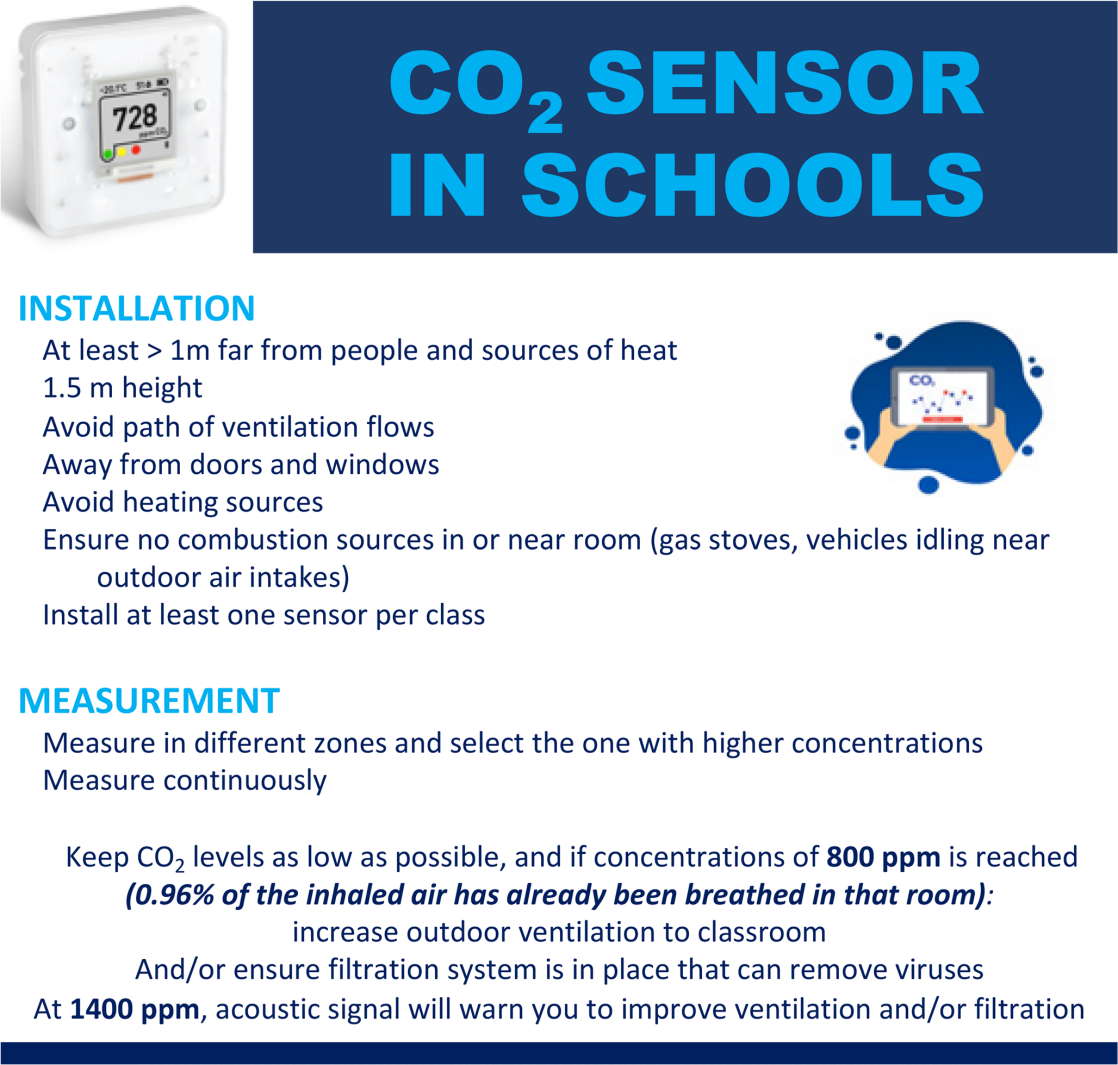
Example of information factsheet at each CO_2_ monitoring station

Along with the monitoring system, we implemented a webpage with project details and aims. We also provided information CO_2_ factsheets at each monitoring location (Fig. 1) along with QR code with link to project online page and real-time measurement levels of PM. These factsheets included a clear outline of the project’s aims and objectives, guidelines for adequate CO_2_ monitoring, and recommended actions based on specific CO_2_ thresholds (at 800 ppm and 1400 ppm). Based on these reports and on the guidelines developed within the AIREAMOS research group (www.aireamos.org), we developed recommendations to enhance ventilation practices and improve indoor air quality in the monitored environments. In particular, indication to increase ventilation (e.g. opening windows) were provided at CO_2_ concentrations above 800 and 1400 ppm, this latter also followed but an acoustic signal indicating threshold passing.

## Results

**Fig. 2:**
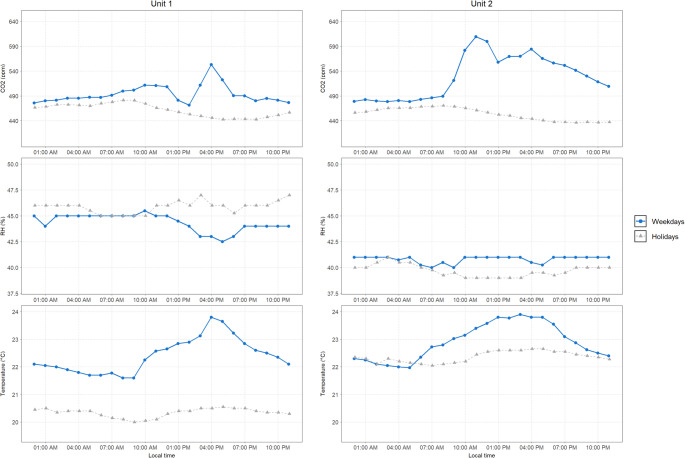
Average hourly values across Unit 1 and Unit 2 during both weekdays and weekends and holidays. Hourly mean values are shown for CO_2_ (ppm), relative humidity (%) and temperature (°C). Solid lines represent weekdays (lecture days) and dashed lines represent weekends and holidays. Data are aggregated across the whole monitoring period for each unit (Unit 1: small, naturally ventilated classroom; Unit 2: large, HVAC-served classroom)

In the Public Health Department classroom (Unit 1), lectures primarily took place in the afternoon, while in the Engineering Department classroom (Unit 2) lectures were held for 8–9 h a day, starting either at 08:00 or 09:00 AM and ending at 06:00 or 07:00 PM, from Monday to Friday. The hourly average values for CO_2_, relative humidity, and temperature in both units during weekdays and weekends and holidays are shown in Fig. 2. Further details on daily average values, including on particulate matter measurements, are provided in Table 2.

**Table 2:** Levels of investigated parameters divided by classroom and types of days

	Weekdays			Weekends and Holidays		
	Mean (SD)	Median (IQR)	Range	Mean (SD)	Median (IQR)	Range
	Unit 1					
CO_2_ (ppm)	561 (231)	482 (458-517)	419–2809	456 (30)	456 (439-477)	401–582
PM_10_ (µg/m^3^)	8.2 (15.9)	4.98 (2.39–9.74)	0.1-1821.7	6.0 (7.0)	3.69 (1.74–7.53)	0.2–76.8
PM_2.5_ (µg/m^3^)	5.7 (5.9)	4.02 (2.19–7.02)	0.1-142.4	4.9 (5.3)	3.29 (1.67–6.01)	0.2–62.0
PM_1_ (µg/m^3^)	3.4 (3.6)	2.26 (1.25-4.00)	0.1–53.0	3.2 (3.6)	1.95 (1.00-3.85)	0.2–31.4
RH (%)	43.7 (10.5)	45.0 (38.0–50.0)	15.0–74.0	40.8 (8.2)	41.0 (26.0–50.0)	20.0–58.0
Temperature (°C)	23.4 (3.6)	24.0 (20.8–27.1)	13.7–32.9	23.2 (4.3)	20.2 (19.4–22.5)	15.4–32.2
Pressure (hPa)	1014 (7)	1014 (1009-1019)	996–1035	1014 (8)	1016 (1013-1022)	993–1035
	Unit 2					
CO_2_ (ppm)	537 (96)	507 (462-588)	410–1131	456 (10)	456 (438-476)	401–582
PM_10_ (µg/m^3^)	5.7 (10.9)	2.73 (1.35–5.94)	0.2–706.0	4.1 (5.0)	2.42 (1.23–4.63)	0.1–55.2
PM_2.5_ (µg/m^3^)	3.3 (3.4)	2.29 (1.25–4.21)	0.2–122.0	3.7 (4.4)	2.24 (1.19–4.03)	0.1–37.5
PM_1_ (µg/m^3^)	2.2 (2.1)	1.46 (0.81–2.74)	0.0-22.1	2.7 (3.3)	1.58 (0.87–2.87)	0.1–24.3
RH (%)	40.8 (7.5)	40.0 (34.0–44.0)	19.0–59.0	40.8 (8.2)	39.0 (34.0–44.0)	20.0–58.0
Temperature (°C)	23.7 (2.9)	22.8 (21.4–24.3)	15.8–31.0	23.2 (4.3)	21.2 (19.1–24.1)	15.4–32.2
Pressure (hPa)	1013 (9)	1013 (1008-1019)	987–1036	1014 (8)	1013 (1009-1019)	993–1035

Average weekday CO_2_ levels were similar across both departments, hovering around 545 ppm – albeit slightly higher and more variable in Unit 1 (561 ppm, standard deviation-SD = 231 ppm) than Unit 2 (537 ppm, SD = 96 ppm). However, a rise in CO_2_ concentrations was observed in both units, starting around 9:00 AM. In Engineering classroom, CO_2_ levels peaked at approximately 610 ppm between 11:00 AM and 12:00 PM, remaining above 550 ppm until 7:00 PM. In Public Health classroom, the mean peak level was slightly lower, going slightly above 540 ppm at 4:00 PM. On weekends and holidays, mean CO_2_ levels in both units dropped, stabilizing around 456 ppm (SD = 30 ppm).

Humidity levels were consistently slightly higher in Public Health classroom, averaging around 45% and dropping to 42.5% during peak occupancy, whereas Engineering classroom rarely exceeded 41.0%, being overall more stable. On weekends and holidays, humidity in Engineering classroom dropped further, while in Public Health classroom it was more variable, often higher than on weekdays. Daily mean values indicate the same trends.

Temperature trends also differed between the two departments. Engineering classroom saw higher average temperatures during weekdays, with a steady rise through the morning that peaked at around 24 °C in the early afternoon, which coincides with peak levels and CO_2_ concentrations. The mean daily temperature was 23.7 °C (SD = 2.9 °C) during weekdays. On weekends, temperatures were more stable, fluctuating less and staying consistently at around 22 °C, lower than the weekday maximums. A similar pattern occurred in the Public Health classroom, where temperatures reached a maximum of almost 24.0 °C at 4:00 PM, again reflecting peak classroom use. Average daily temperature was 23.4 °C (SD = 3.7 °C). On weekends and holidays, temperatures in Public Health classroom were lower and more consistent, dropping to a minimum of 20.0 °C.

CO_2_ levels showed higher variability between the two departments when looking at individual measurements (Supplementary Fig. S1). In Engineering classroom, the highest recorded value was close to 1130 ppm. In contrast, Public Health classroom consistently recorded levels higher than this, especially from late April to mid-May and again from September onward. Despite some interruptions in data collection, Public Health classroom showed a more fluctuating pattern, with the highest recorded value reaching 2809 ppm in mid-November, a period that saw the highest overall CO_2_ levels in the classroom.

**Fig. 3:**
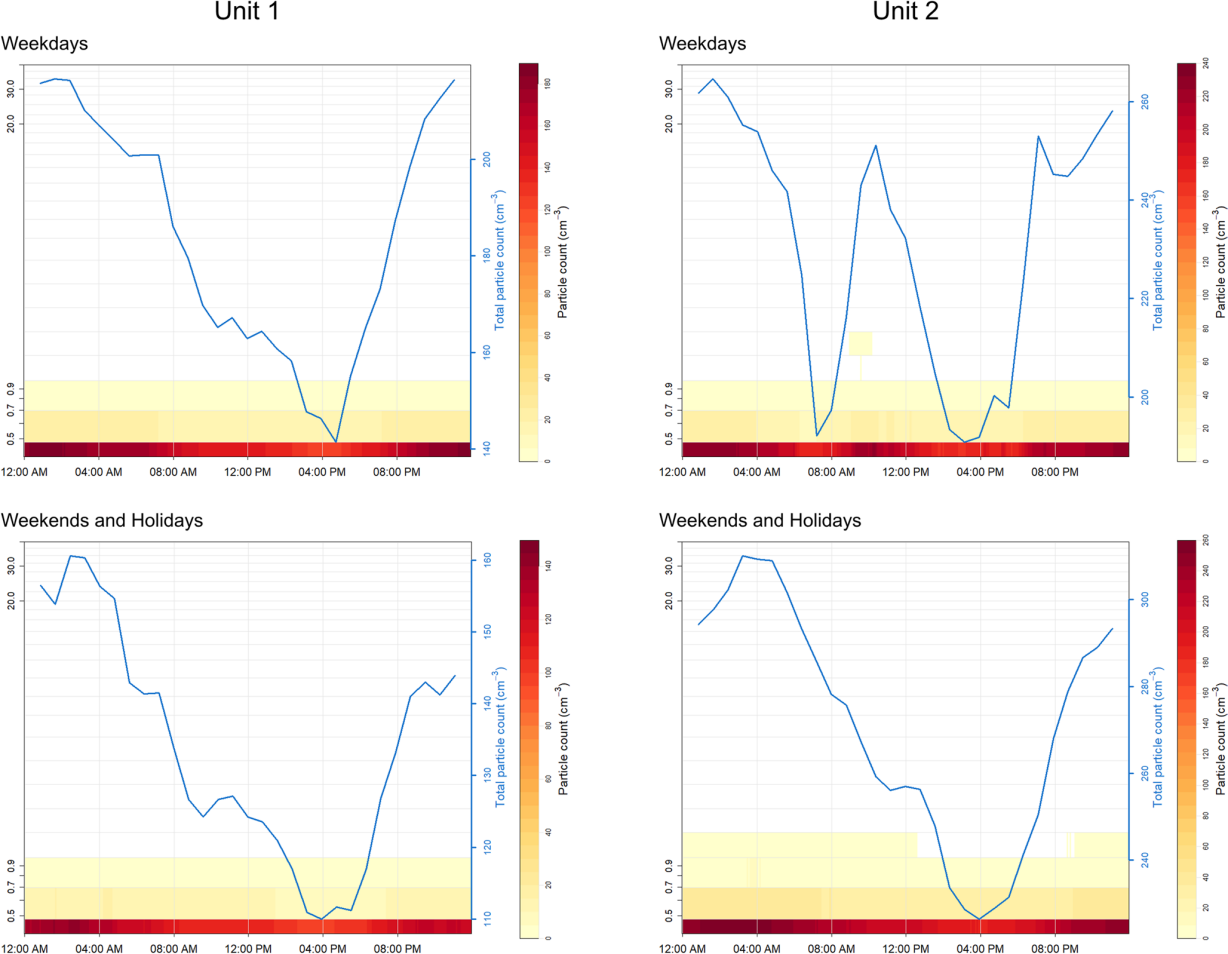
Daily trends of total particle count and particle concentration during weekdays. Hourly aggregated values during weekdays for Unit 1 (small, naturally ventilated classroom) and Unit 2 (large, HVAC-served classroom). Total particle count is expressed as number of particles per cm^3^; particle concentrations are expressed as µg/m^3^ (PM_1_, PM_2.5_, PM_10_) derived from the OPC-N3 size distribution

The Public Health classroom consistently showed higher PM_10_ levels and larger variability during weekdays, with a mean of 8.2 µg/m^3^ (SD = 15.9 µg/m^3^), compared to 5.7 µg/m^3^ (SD = 10.9 µg/m^3^) in the Engineering classroom (Fig. 3). On weekends and holidays, mean PM_10_ levels and most of their variability decreased across both units, with Public Health classroom recording 6.0 µg/m^3^ (SD = 7.0 µg/m^3^) and Engineering classroom recording 4.1 µg/m^3^ (SD = 5.0 µg/m^3^).

PM_2.5_ and PM_1_ concentrations followed a similar trend, with higher values recorded in Public Health classroom during weekdays (mean PM_2.5_ = 5.7 µg/m^3^, SD = 5.9 µg/m^3^; mean PM_1_ = 3.4 µg/m^3^, SD = 3.6 µg/m^3^) compared to Engineering classroom (mean PM_2.5_ = 3.3 µg/m^3^, SD = 3.4 µg/m^3^; mean PM_1_ = 2.2 µg/m^3^, SD = 2.1 µg/m^3^). On weekdays, total particle count in Unit 1 gradually decreases from early morning, reaching a minimum between 12:00 PM and 4:00 PM. Afterward, particle count increases sharply, peaking in the late afternoon. On weekends and holidays, total particle concentration exhibits a similar pattern to weekdays but with more gradual fluctuations throughout the day. In Unit 2, on weekdays, the diurnal trend of total particle count shows multiple peaks, with an initial decrease early in the morning, then a sharp increase around 09:00 AM, reaching peak concentration between 10:00 AM and 12:00 PM. A second drop occurs in the early afternoon, followed by a rapid rise around 4:00 PM. On weekends and holidays, the trend in Unit 2 mirrors the behavior observed in Unit 1: total particle concentration remains low and stable throughout the day, with more moderate fluctuations.

The total particulate count, despite occasional interruption in measurements, displayed a clear seasonal trend in both units, with higher values observed during the winter months (Supplementary Fig. S2). In Unit 1, peaks occurred between September and October, December and January, and February, which recorded the highest count. Similarly, Unit 2 displayed an analogous pattern, with multiple peaks throughout the winter and a maximum value recorded between December and January.

## Discussion

In this study, we conducted continuous indoor air quality (IAQ) monitoring in two distinct classrooms: one (Unit 1) smaller, relying on natural ventilation and wall radiators, and the other (Unit 2) larger, featuring HVAC system and regular schedule of teaching hours. The monitoring was carried out cost-effectively, using accessible yet highly reliable sensors, and the collected data was systematically analyzed to uncover trends and differences in air quality between the two environments.

As indoor CO_2_ primarily comes from the occupants of the building and it is removed by ventilation [35], CO_2_ concentrations are considered an effective proxy for ventilation rates [36] and are therefore considered instrumental not only in identifying spaces with suboptimal air exchange, but also to provide insights into the control of other indoor-generated pollutants and aerosols [37]. Inadequate ventilation that relies on recirculated unfiltered air, rather than fresh outdoor air, can increase the potential for airborne disease spread if an infected person is present [38], with high CO_2_ levels correlating with higher likelihood of infection [39, 40]. The slightly higher average CO_2_ levels demonstrated in Unit 1 compared to Unit 2 (561 vs. 537 ppm) can be attributed to its smaller size and reliance on natural ventilation via windows and a single door, a setup that leads to less efficient air exchange, especially when occupancy increases. The room occupancy and characteristics also explain its greater diurnal fluctuations, as CO_2_ levels spike more easily in such an environment when windows are not consistently opened or when the classroom is frequently at full capacity [41]. In contrast, Unit 2 benefits from an HVAC system and greater spatial volume, which help dilute CO_2_ and provide more stable air exchange, despite accommodating a higher number of students (up to 228 compared to 25 in Unit 1).

In one study carried out on Italian primary schools relying on natural ventilation, CO_2_ levels were notably higher than those of our Units, ranging from 782 to 4064 ppm, likely due to the smaller sizes of the classes investigated and the generally long and permanent occupancy by the same students for the entire morning [42]. This finding was also confirmed by another Italian study reporting a clear relationship between classroom size, occupancy density, and CO_2_ levels, with larger or less crowded rooms showing improved air quality [43].

The lower CO_2_ levels in university classrooms compared to other school grades (primary, middle and high schools) confirm also the lower risk of COVID-19 infection through airborne transmission in the first setting as evidenced in a review compared the likelihood of COVID-19 infection in different settings, including home, restaurants and concert halls, finding it low in college classrooms [44]. However, relying exclusively on CO_2_ concentration targets to assess or reduce infection risks indoors is likely inadequate, as infection-related CO_2_ thresholds can differ by more than two orders of magnitude depending on the setting and activities involved [40]. For this reason, we integrated two sensors into each measurement location to capture also particulate matter along with CO_2_.

This strategy using OPC-N3 sensors have been successfully used within indoor air quality studies, including domestic aerosol levels during the COVID-19 lockdown [45] or within a urban cohort study on 72 households [46]. As a matter of that, indoor air quality monitoring of PM levels are equally important, directly affecting respiratory and cardiovascular health [47–49]. Specifically, both acute and long-term exposure to PM_2.5_ and PM_10_, particles with diameters under 2.5 μm and 10 μm, respectively, have been associated with increased risks of airway inflammation, asthma exacerbation, ischemic heart disease, and poorer academic performance in younger populations [50, 51]. The most common sources of PM include emissions from fuel combustion from nearby roads, construction sites, and natural sources, such as dust from the outdoors or pollen [52]. The most recent WHO air quality guidelines on particulate matter do not differentiated between indoor and outdoor levels [53], setting the upper limit of PM_2.5_ concentrations at 25 µg/m^3^ for 24-hour and 10 µg/m^3^ for 1-year averages. In our study, average PM_10_ levels were higher on weekdays in Unit 1 compared to Unit 2 (8.2 µg/m^3^ vs. 5.7 µg/m^3^), likely due to the limited ventilation and potential particle resuspension from student activity in the smaller space. Both units exhibited higher PM concentrations during the winter months, particularly in December and January. This seasonal pattern can be attributed mainly to reduced ventilation during colder months, leading to particle accumulation, and to an increased use of heating systems, which may contribute to particle movement. Additionally, levels of outdoor PM levels due to motorized traffic and heating systems are generally higher in cold seasons [54], thus contributing to higher levels of PM indoor. Despite such variations, in both units the average values consistently remained within the guideline limits, with limited and occasional spikes likely caused by full room occupation, ventilation patterns and the movement of people inside the space. This issue suggests that placement of sensors should be carefully evaluated to avoid or at least limit the influence of these factors in measurements [45].

Relative humidity (RH) and temperature also play crucial roles in maintaining a healthy indoor environment. RH affects not only occupant comfort but also the viability of airborne pathogens and the resuspension of particulate matter [55]. Furthermore, levels outside the optimal range of 40–60% can exacerbate respiratory issues if lower (e.g., reduced mucociliary clearance efficiency) and encourage the proliferation of mold and other allergens if higher [56, 57]. In our classes, both units showed levels within this range, with Unit 1 showing higher average humidity levels compared to Unit 2 (45.0% vs. 41.0%). The higher relative humidity in Unit 1 over weekends can be attributed to the radiators being turned off at night and during weekends, which also explains the lower temperatures observed on Monday mornings. Nonetheless, the overall trends of higher RH in Unit 1 and more stable lower values in Unit 2 persisted across daily mean values, further confirm that ventilation type and heating systems have high and relevant impact on moisture control. Despite this, both units successfully demonstrated RH levels within the ideal range considered ideal to reduce acute symptoms, improve productivity, and lower the likelihood of respiratory infections [56].

Similarly, temperature impacts occupant well-being and cognitive performance while influencing ventilation efficiency and pollutant behavior: high temperatures can enhance the off-gassing of volatile organic compounds (VOCs), while cooler temperatures may compromise thermal comfort and discourage window ventilation [58, 59]. Exposure to excessive cold or excessive heat indoors are both associated with negative health effects, such as increases in blood pressure, ischemic stroke or heat exhaustion [60, 61]. In addition, low temperature has been shown to increase both viability and transmission of viruses, while high temperatures are linked to smaller and lighter aerosols [62, 63]. There is also an interplay between temperature and RH to consider, as a cold and dry air impairs mucus secretion, leading to a less efficient immune response [64]. In our data, weekday temperatures in Unit 2 averaged 23.7 °C, with a steady rise through the morning, peaking at approximately 24 °C in the early afternoon. This coincided with peak CO_2_ levels and occupancy. In Unit 1, temperatures followed a similar pattern, peaking at almost 24 °C by 04:00 PM, aligning with peak classroom usage. The wall radiators in Unit 1 being turned off during weekends and holidays resulted in lower temperatures during these periods. This effect was less pronounced in Unit 2 due to the continuous operation of the HVAC system, maintaining consistent temperatures regardless of occupancy. In any case, and despite their differences, both units managed to preserve temperatures in the optimal range during their usage.

There are some limitations to consider. First, guidelines and protocols for CO_2_ monitoring in indoor environments, especially classrooms, are inconsistent and vary substantially [65], with some studies suggesting that a single measurement point may be sufficient for rooms with good ventilation and stable occupancy [66]. Given the distinct characteristics of our units (such as size and ventilation system), more than one sensor per room could have been required, placed in different locations and at various heights, and considering the sensor’s placement relative to doors and windows. In any case, a single sensor was likely sufficient for Unit 1, despite its natural ventilation, while for Unit 2, given its size, two sensors might have improved accuracy. The reason is that CO_2_ is a gas that tends to be unevenly distributed across the space and greatly affected by occupancy [67, 68], making the selection of an appropriate number of measurement points and their location a potential bias during the data gathering phase. An additional improvement could have been the inclusion of an outdoor monitoring station to provide a basis for comparing indoor values with outdoor trends [40], as the apportionment of indoor PM into infiltrated outdoor particles versus indoor-generated particles was not possible. This is also one of the reasons the analysis was primarily descriptive: with only two units, formal between-room statistical comparisons could have been driven by building-specific confounders rather than by ventilation per se, and within-room temporal models would have required outdoor exposure data and/or individual-level occupancy.

Although we framed CO_2_ and PM as informative proxy indicators of ventilation adequacy and of the corresponding potential for airborne transmission, this study did not include direct measurement of infection outcomes nor the application of quantitative risk-assessment frameworks such as the Wells-Riley equation or the rebreathed-fraction model [69], as the reporting of these models calculations under such uncertainty would have largely reproduced the wide range of literature estimates rather than added information on these specific classrooms.

Additionally, on several occasions, the sensors stopped recording, requiring manual intervention to restore functionality, which resulted in some data gaps, although this issue did not affect the average measurements. It is worth noting that the gaps were not uniformly distributed: in Unit 1 the longer interruption of the OPC-N3 measurements occurred predominantly between late spring and early summer, that is, after the end of the teaching semester (late May), so it is unlikely to bias the estimates of mean occupancy-driven PM levels; in contrast, CO_2_ missingness was characterized by more frequent but generally shorter interruptions distributed throughout the monitoring period: 19 interruptions in Unit 1, and 25 interruptions in Unit 2. This pattern suggests, at most, a limited potential for bias in daily and diurnal averages.

This small sample size limits the external validity of our results, which should not be extrapolated uncritically to different building types, climates, or educational levels. We nevertheless argue that the approach is generalizable to other settings provided that the sensor deployment is paired with adequate occupant communication and continuous training on indoor air quality.

In addition to these limitations, this study has some strengths. First, continuous measurements of multiple parameters were conducted over an entire year, carefully accounting for periods of classroom activity and inactivity. In addition, the inclusion of classrooms with differing structural characteristics allowed us to evaluate their impact on indoor air quality. Variations in CO_2_ and PM levels between the two monitored classrooms highlight exactly how different building design, ventilation strategies, and occupancy dynamics interplay with each other: Unit 2, with its centralized HVAC, maintained more stable IAQ metrics, yet it was still vulnerable to spikes in CO_2_ during peak occupancy. Conversely, Unit 1 exhibited greater variability and higher peaks, driven largely by its reliance on manual ventilation practices.

Our findings underline the importance of continuous indoor air quality monitoring as a foundation for creating and maintaining a healthy learning environment in universities [13] and further strengthen the need to implement effective education. In our study, each monitoring station featured a factsheet with a QR code linking to the webpage of the project, where students could monitor PM and CO_2_ levels in real time and learn how to respond if limits were exceeded. This proactive method not only helps to maintain a healthier indoor environment but also plays a potential role in preventing the spread of airborne diseases, making the sensors we employed an affordable and useful tool to prevent infectious diseases and more generally to endure high indoor air quality. Ideally, classrooms with high occupancy or specific challenges (such as reliance on natural ventilation with few windows) should be equipped with these cost-effective and user-friendly sensors to encourage active risk reduction measures. On this note, our experience with the communication programme provided an important lesson on the translatability of low-cost sensor deployments to the wider built environment. The factsheet and the QR-coded webpage were a necessary, but likely insufficient component: in Unit 1, despite the displayed 800 ppm and 1400 ppm thresholds and the acoustic signal at 1400 ppm, occupants typically did not act on the signal during the lecture, with windows often left closed until concentrations had reached approximately 2000 ppm or until the lecture ended. This pattern is consistent with what intervention-based studies: real-time visual and acoustic feedback can yield reductions of about 20% in classroom CO_2_ when teachers are explicitly engaged in the response loop, but the magnitude of the effect is conditional on the behavioral component being actively managed [30]. We therefore recommend that future deployments pair the static information material with continuous, lecturer-targeted training emphasizing that ventilation must be increased immediately upon threshold activation rather than at the next break, and – when not feasible – to employ other mitigation strategies, e.g., redistributing classes across larger or better ventilated rooms, or staggering attendance to reduce peak occupancy [26].

Our results, while consistent with recent post-COVID Italian studies in higher-education settings that report substantial seasonal CO_2_ excursions in autumn and winter [70], and with broader school-based work showing that prolonged exposure in shared environments [25], further indicate that low-cost CO_2_ and PM monitoring is not only technically straightforward, but also reliable and easily scalable, including beyond classrooms to other multi-occupancy settings such as kindergartens, healthcare waiting areas, public transportations and offices [71, 72].

## Supplementary Information

Below is the link to the electronic supplementary material.

Supplementary Material 1

## Data Availability

The data that support the findings of the study are available in the article.

## References

[1] Koivisto AJ, Kling KI, Hanninen O, Jayjock M, Londahl J, Wierzbicka A, et al. Source specific exposure and risk assessment for indoor aerosols. Sci Total Environ. 2019;668:13–24. 10.1016/j.scitotenv.2019.02.398. 30851679 10.1016/j.scitotenv.2019.02.398

[2] Wolkoff P. Indoor air humidity, air quality, and health - An overview. Int J Hyg Environ Health. 2018;221(3):376–90. 10.1016/j.ijheh.2018.01.015. 29398406 10.1016/j.ijheh.2018.01.015

[3] Alhorr Y, Arif M, Katafygiotou M, Mazroei A, Kaushik A, Elsarrag E. Impact of indoor environmental quality on occupant well-being and comfort: a review of the literature. Int J Sustain Built Environ. 2016;5(1):1–11. 10.1016/j.ijsbe.2016.03.006.

[4] Graudenz GS, Latorre MR, Tribess A, Oliveira CH, Kalil J. Persistent allergic rhinitis and indoor air quality perception–an experimental approach. Indoor Air. 2006;16(4):313–9. 10.1111/j.1600-0668.2006.00428.x. 16842611 10.1111/j.1600-0668.2006.00428.x

[5] Rollins SM, Su FC, Liang X, Humann MJ, Stefaniak AB, LeBouf RF, et al. Workplace indoor environmental quality and asthma-related outcomes in healthcare workers. Am J Ind Med. 2020;63(5):417–28. 10.1002/ajim.23101. 32154609 10.1002/ajim.23101 PMC7437141

[6] Zhang X, Li D, Xie J, Liu J. Environmental perceptions, mental performance, and physiological responses of people with respiratory allergies exposed to reduced Indoor Air Quality. Indoor Air. 2021;31(5):1458–72. 10.1111/ina.12793. 33432603 10.1111/ina.12793

[7] Riva A, Rebecchi A, Capolongo S, Gola M. Can homes affect well-Being? A scoping review among housing conditions, indoor environmental quality, and mental health outcomes. Int J Environ Res Public Health. 2022;19(23):15975. 10.3390/ijerph192315975. 36498051 10.3390/ijerph192315975 PMC9736414

[8] Wei J, Li Y. Airborne spread of infectious agents in the indoor environment. Am J Infect Control. 2016;44(9 Suppl):S102–8. 10.1016/j.ajic.2016.06.003. 27590694 10.1016/j.ajic.2016.06.003 PMC7115322

[9] Vinceti M, Balboni E, Rothman KJ, Teggi S, Bellino S, Pezzotti P, et al. Substantial impact of mobility restrictions on reducing COVID-19 incidence in Italy in 2020. J Travel Med. 2022;29(6). 10.1093/jtm/taac081. 10.1093/jtm/taac081 PMC9384467 35876268

[10] Argyropoulos CD, Skoulou V, Efthimiou G, Michopoulos AK. Airborne transmission of biological agents within the indoor built environment: a multidisciplinary review. Air Qual Atmos Health. 2023;16(3):477–533. 10.1007/s11869-022-01286-w. 36467894 10.1007/s11869-022-01286-w PMC9703444

[11] Lu CY, Tsai MC, Muo CH, Kuo YH, Sung FC, Wu CC. Personal, psychosocial and environmental factors related to sick building syndrome in official employees of Taiwan. Int J Environ Res Public Health. 2017;15(1):7. 10.3390/ijerph15010007. 29271881 10.3390/ijerph15010007 PMC5800107

[12] Azuma K, Yanagi U, Kagi N, Kim H, Ogata M, Hayashi M. Environmental factors involved in SARS-CoV-2 transmission: effect and role of indoor environmental quality in the strategy for COVID-19 infection control. Environ Health Prev Med. 2020;25(1):66. 10.1186/s12199-020-00904-2. 33143660 10.1186/s12199-020-00904-2 PMC7607900

[13] D’Alessandro D, Rebecchi A, Appolloni L, Brambilla A, Brusaferro S, Buffoli M, et al. Re-thinking the environment, cities, and living spaces for public health purposes, according with the COVID-19 lesson: the LVII Erice Charter. Land. 2023;12(10):1863. 10.3390/land12101863.

[14] Di Gilio A, Palmisani J, Pulimeno M, Cerino F, Cacace M, Miani A, et al. CO(2) concentration monitoring inside educational buildings as a strategic tool to reduce the risk of Sars-CoV-2 airborne transmission. Environ Res. 2021;202:111560. 10.1016/j.envres.2021.111560. 34224708 10.1016/j.envres.2021.111560 PMC8253691

[15] Saini J, Dutta M, Marques G. Sensors for indoor air quality monitoring and assessment through Internet of Things: a systematic review. Environ Monit Assess. 2021;193(2):66. 10.1007/s10661-020-08781-6. 33452599 10.1007/s10661-020-08781-6

[16] Kim KN, Kim S, Lim YH, Song IG, Hong YC. Effects of short-term fine particulate matter exposure on acute respiratory infection in children. Int J Hyg Environ Health. 2020;229:113571. 10.1016/j.ijheh.2020.113571. 32554254 10.1016/j.ijheh.2020.113571

[17] Landrigan PJ, Fuller R, Acosta NJR, Adeyi O, Arnold R, Basu NN, et al. The Lancet Commission on pollution and health. Lancet. 2018;391(10119):462–512. 10.1016/S0140-6736(17)32345-0. 29056410 10.1016/S0140-6736(17)32345-0

[18] Cohen AJ, Brauer M, Burnett R, Anderson HR, Frostad J, Estep K, et al. Estimates and 25-year trends of the global burden of disease attributable to ambient air pollution: an analysis of data from the Global Burden of Diseases Study 2015. Lancet. 2017;389(10082):1907–18. 10.1016/S0140-6736(17)30505-6. 10.1016/S0140-6736(17)30505-6 PMC5439030 28408086

[19] Chang S, Pierson E, Koh PW, Gerardin J, Redbird B, Grusky D, et al. Mobility network models of COVID-19 explain inequities and inform reopening. Nature. 2021;589(7840):82–7. 10.1038/s41586-020-2923-3. 33171481 10.1038/s41586-020-2923-3

[20] Paduano S, Facchini MC, Greco A, Borsari L, Mingrone VM, Tancredi S, et al. Characteristics and risk factors of isolated and quarantined children and adolescents during the first wave of SARS-CoV-2 pandemic: a cross-sectional study in Modena, Northern Italy. Acta Biomed. 2021;92(S6):e2021449. 10.23750/abm.v92iS6.12225. 34739471 10.23750/abm.v92iS6.12225 PMC8850999

[21] Braggion A, Dugerdil A, Wilson O, Hovagemyan F, Flahault A. Indoor Air Quality and COVID-19: a scoping review. Public Health Rev. 2023;44:1605803. 10.3389/phrs.2023.1605803. 38273885 10.3389/phrs.2023.1605803 PMC10810127

[22] Rodriguez D, Urbieta IR, Velasco A, Campano-Laborda MA, Jimenez E. Assessment of indoor air quality and risk of COVID-19 infection in Spanish secondary school and university classrooms. Build Environ. 2022;226:109717. 10.1016/j.buildenv.2022.109717. 36313012 10.1016/j.buildenv.2022.109717 PMC9595429

[23] D’Alessandro D, Gola M, Appolloni L, Dettori M, Fara GM, Rebecchi A, et al. COVID-19 and living space challenge. Well-being and public health recommendations for a healthy, safe, and sustainable housing. Acta Biomed. 2020;91(9–S):61–75. 10.23750/abm.v91i9-S.10115. 10.23750/abm.v91i9-S.10115 PMC8023091 32701918

[24] Gola M, Caggiano G, De Giglio O, Napoli C, Diella G, Carlucci M, et al. SARS-CoV-2 indoor contamination: considerations on anti-COVID-19 management of ventilation systems, and finishing materials in healthcare facilities. Ann Ig. 2021;33(4):381–92. 10.7416/ai.2020.2396. 33270076 10.7416/ai.2020.2396

[25] Banholzer N, Munday JD, Jent P, Bittel P, Dall’Amico L, Furrer L, et al. The relative contribution of close-proximity contacts, shared classroom exposure and indoor air quality to respiratory virus transmission in schools. Nat Commun. 2025;16(1):11678. 10.1038/s41467-025-66719-3. 41309642 10.1038/s41467-025-66719-3 PMC12749238

[26] Shoubridge AP, Brass A, Elms L, Sims SK, Anderson A, Mordaunt D, et al. Atmospheric CO(2) monitoring to identify zones of increased airborne pathogen transmission risk in hospital settings. Am J Infect Control. 2025;53(2):266–8. 10.1016/j.ajic.2024.10.001. 39369823 10.1016/j.ajic.2024.10.001

[27] Banholzer N, Bittel P, Jent P, Furrer L, Zurcher K, Egger M, et al. Molecular detection of SARS-CoV-2 and other respiratory viruses in saliva and classroom air: a two winters tale. Clin Microbiol Infect. 2024;30(6):e8291–4. 10.1016/j.cmi.2024.03.002. 10.1016/j.cmi.2024.03.002 38467247

[28] Sun Y, Haghnazari D, Huang CY, Baig A, Kim M, Cunningham A, et al. Air purifier intervention for respiratory viral exposure in elementary schools: a secondary analysis of a randomized clinical trial. JAMA Netw Open. 2025;8(10):e2536951. 10.1001/jamanetworkopen.2025.36951. 41071551 10.1001/jamanetworkopen.2025.36951 PMC12514627

[29] Park S, Choi Y, Song D, Kim EK. Natural ventilation strategy and related issues to prevent coronavirus disease 2019 (COVID-19) airborne transmission in a school building. Sci Total Environ. 2021;789:147764. 10.1016/j.scitotenv.2021.147764. 34051507 10.1016/j.scitotenv.2021.147764 PMC8123370

[30] Rawat N, Kumar P, Hama S, Williams N, Zivelonghi A. Improving classroom air quality and ventilation with IoT-driven acoustic and visual CO(2) feedback system. Sci Total Environ. 2025;980:179543. 10.1016/j.scitotenv.2025.179543. 40318364 10.1016/j.scitotenv.2025.179543

[31] Finlayson-Pitts BJ, Pitts JN. Chemistry of the upper and lower atmosphere theory, experiments, and applications. In: Finlayson-Pitts BJ, Pitts JN, editors. Chemistry of the Upper and Lower Atmosphere. San Diego: Academic; 2000. pp. 547–656.

[32] Villanueva F, Jiménez E, Felisi JM, Garrido T, Jiménez JL, Ródenas M et al. Guide about affordable CO2 detectors for COVID-19 Prevention. https://bit.ly/monitorsCO2 (2021). Accessed 10/12/2024.

[33] Sorensen CM, Gebhart J, O’Hern TJ, Rader DJ. Optical measurement techniques: fundamentals and applications. In: Kulkarni P, Baron PA, Willeke K, editors. Aerosol measurement: principles, techniques, and applications. John Wiley & Sons, Inc.; 2011. pp. 269–312.

[34] Alfano B, Barretta L, Del Giudice A, De Vito S, Di Francia G, Esposito E, et al. A review of low-cost particulate matter sensors from the developers’ perspectives. Sens (Basel). 2020;20(23):6819. 10.3390/s20236819. 10.3390/s20236819 PMC7730878 33260320

[35] Persily A. Development and application of an indoor carbon dioxide metric. Indoor Air. 2022;32(7):e13059. 10.1111/ina.13059. 35904382 10.1111/ina.13059

[36] EnvironmentalModelling Group (EMG), Scientific Pandemic Insights Group on Behaviours (SPI-B). Application of CO2 monitoring as an approach to managing ventilation to mitigate SARS-CoV-2 transmission. UK, 2021. https://www.gov.uk/government/publications/emg-and-spi-b-application-of-co2-monitoring-as-an-approach-to-managing-ventilation-to-mitigate-sars-cov-2-transmission-27-may-2021. Accessed 12 Oct 2024.

[37] Kurnitski J, Kiil M, Wargocki P, Boerstra A, Seppanen O, Olesen B, et al. Respiratory infection risk-based ventilation design method. Build Environ. 2021;206:108387. 10.1016/j.buildenv.2021.108387. 34602721 10.1016/j.buildenv.2021.108387 PMC8462055

[38] Taylor JG, Yates TA, Mthethwa M, Tanser F, Abubakar I, Altamirano H. Measuring ventilation and modelling *M. tuberculosis* transmission in indoor congregate settings, rural KwaZulu-Natal. Int J Tuberc Lung Dis. 2016;20(9):1155–61. 10.5588/ijtld.16.0085. 27510239 10.5588/ijtld.16.0085 PMC4978153

[39] Li Y, Leung GM, Tang JW, Yang X, Chao CY, Lin JZ, et al. Role of ventilation in airborne transmission of infectious agents in the built environment - a multidisciplinary systematic review. Indoor Air. 2007;17(1):2–18. 10.1111/j.1600-0668.2006.00445.x. 17257148 10.1111/j.1600-0668.2006.00445.x

[40] Peng Z, Jimenez JL, Exhaled. CO(2) as a COVID-19 infection risk proxy for different indoor environments and activities. Environ Sci Technol Lett. 2021;8(5):392–7. 10.1021/acs.estlett.1c00183. 37566374 10.1021/acs.estlett.1c00183

[41] Zhang H, Srinivasan R, Yang X, Ganesan V, Zhang H. Diurnal variation of indoor air pollutants and their influencing factors in educational buildings: a case study using LASSO-based ANNs. Atmos Environ. 2024;333:120673. 10.1016/j.atmosenv.2024.120673.

[42] Stabile L, Massimo A, Canale L, Russi A, Andrade A, Dell’Isola M. The effect of ventilation strategies on indoor air quality and energy consumptions in classrooms. Buildings. 2019;9(5):110. 10.3390/buildings9050110.

[43] Babich F, Torriani G, Corona J, Lara-Ibeas I. Comparison of indoor air quality and thermal comfort standards and variations in exceedance for school buildings. J Build Eng. 2023;71:106405. 10.1016/j.jobe.2023.106405.

[44] Iwamura N, Tsutsumi K, Hamashoji T, Arita Y, Deguchi T. Carbon dioxide levels as a key indicator for managing SARS-CoV-2 airborne transmission risks across 10 indoor scenarios. Cureus. 2024;16(11):e74429. 10.7759/cureus.74429. 39600549 10.7759/cureus.74429 PMC11590689

[45] Kaliszewski M, Wlodarski M, Mlynczak J, Kopczynski K. Comparison of low-cost particulate matter sensors for indoor air monitoring during COVID-19 lockdown. Sens (Basel). 2020;20(24):7290. 10.3390/s20247290. 10.3390/s20247290 PMC7766947 33353048

[46] Chu MT, Gillooly SE, Levy JI, Vallarino J, Reyna LN, Cedeno Laurent JG, et al. Real-time indoor PM(2.5) monitoring in an urban cohort: implications for exposure disparities and source control. Environ Res. 2021;193:110561. 10.1016/j.envres.2020.110561. 33275921 10.1016/j.envres.2020.110561 PMC7856294

[47] Adams K, Greenbaum DS, Shaikh R, van Erp AM, Russell AG. Particulate matter components, sources, and health: systematic approaches to testing effects. J Air Waste Manag Assoc. 2015;65(5):544–58. 10.1080/10962247.2014.1001884. 25947313 10.1080/10962247.2014.1001884

[48] Sciannameo V, Goffi A, Maffeis G, Gianfreda R, Jahier Pagliari D, Filippini T, et al. A deep learning approach for spatio-temporal forecasting of new cases and new hospital admissions of COVID-19 spread in Reggio Emilia, Northern Italy. J Biomed Inf. 2022;132:104132. 10.1016/j.jbi.2022.104132. 10.1016/j.jbi.2022.104132 PMC9271423 35835438

[49] Chen J, Rodopoulou S, de Hoogh K, Strak M, Andersen ZJ, Atkinson R, et al. Long-term exposure to fine particle elemental components and natural and cause-specific mortality-a pooled analysis of eight European cohorts within the ELAPSE Project. Environ Health Perspect. 2021;129(4):47009. 10.1289/EHP8368. 33844598 10.1289/EHP8368 PMC8041432

[50] Branco P, Alvim-Ferraz MCM, Martins FG, Ferraz C, Vaz LG, Sousa SIV. Impact of indoor air pollution in nursery and primary schools on childhood asthma. Sci Total Environ. 2020;745:140982. 10.1016/j.scitotenv.2020.140982. 32736106 10.1016/j.scitotenv.2020.140982

[51] Sadrizadeh S, Yao R, Yuan F, Awbi H, Bahnfleth W, Bi Y, et al. Indoor air quality and health in schools: a critical review for developing the roadmap for the future school environment. J Build Eng. 2022;57:104908. 10.1016/j.jobe.2022.104908.

[52] EEA.Europe’s air quality status. Europe. 2022. https://www.eea.europa.eu/en/analysis/publications/air-quality-in-europe-2022. Accessed 12 Oct 2024.

[53] WHO. WHO global air quality guidelines: particulate matter (PM2. 5 and PM10), ozone, nitrogen dioxide, sulfur dioxide and carbon monoxide. World Health Organization; 2021. 34662007

[54] Pietrodangelo A, Bove MC, Forello AC, Crova F, Bigi A, Brattich E, et al. A PM10 chemically characterized nation-wide dataset for Italy. Geographical influence on urban air pollution and source apportionment. Sci Total Environ. 2024;908:167891. 10.1016/j.scitotenv.2023.167891. 37852492 10.1016/j.scitotenv.2023.167891

[55] Si X, Mengersen K, Ye C, Hu W. Interactive effect of air pollutant and meteorological factors on seasonal influenza transmission, Shanghai, China. Atmos Environ. 2024;318:120208. 10.1016/j.atmosenv.2023.120208.

[56] Wolkoff P. Indoor air humidity revisited: Impact on acute symptoms, work productivity, and risk of influenza and COVID-19 infection. Int J Hyg Environ Health. 2024;256:114313. 10.1016/j.ijheh.2023.114313. 38154254 10.1016/j.ijheh.2023.114313

[57] Pal R, Sarkar S, Mukhopadhyay A. Influence of ambient conditions on evaporation and transport of respiratory droplets in indoor environment. Int Commun Heat Mass Transf. 2021;129:105750. 10.1016/j.icheatmasstransfer.2021.105750.

[58] Felgueiras F, Mourão Z, Moreira A, Gabriel MF. Indoor environmental quality in offices and risk of health and productivity complaints at work: a literature review. J Hazard Mater Adv. 2023;10:100314. 10.1016/j.hazadv.2023.100314.

[59] Zhang F, de Dear R, Hancock P. Effects of moderate thermal environments on cognitive performance: a multidisciplinary review. Appl Energy. 2019;236:760–77. 10.1016/j.apenergy.2018.12.005.

[60] Chen X, Tu P, Sun XL, Hu TY, Wan J, Hu YW, et al. The impact on blood pressure of a short-term change in indoor temperature. Int J Gen Med. 2021;14:1507–11. 10.2147/IJGM.S291431. 33911895 10.2147/IJGM.S291431 PMC8075305

[61] Danh N, Ho C, Ford E, Zhang J, Hong H, Reid C, et al. Association between ambient temperature and stroke risk in high-risk populations: a systematic review. Front Neurol. 2023;14:1323224. 10.3389/fneur.2023.1323224. 38259643 10.3389/fneur.2023.1323224 PMC10801432

[62] Kwak D-B, Fischer HD, Pui DY. Saliva droplet evaporation experiment and simple correlation of evaporation-falling curve under different temperatures and RH. Aerosol Air Qual Res. 2023;23(3):220409. 10.4209/aaqr.220409.

[63] Balboni E, Filippini T, Rothman KJ, Costanzini S, Bellino S, Pezzotti P, et al. The influence of meteorological factors on COVID-19 spread in Italy during the first and second wave. Environ Res. 2023;228:115796. 10.1016/j.envres.2023.115796. 37019296 10.1016/j.envres.2023.115796 PMC10069087

[64] Huang D, Taha MS, Nocera AL, Workman AD, Amiji MM, Bleier BS. Cold exposure impairs extracellular vesicle swarm–mediated nasal antiviral immunity. J Allergy Clin Immunol. 2023;151(2):509–25. 10.1016/j.jaci.2022.09.037. e8. 36494212 10.1016/j.jaci.2022.09.037 PMC13380700

[65] Zhang D, Ding E, Bluyssen PM. Guidance to assess ventilation performance of a classroom based on CO2 monitoring. Indoor Built Environ. 2022;31(4):1107–26. 10.1177/1420326x211058743.

[66] Rackes A, Ben-David T, Waring MS. Sensor networks for routine indoor air quality monitoring in buildings: impacts of placement, accuracy, and number of sensors. Sci Technol built Environ. 2018;24(2):188–97. 10.1080/23744731.2017.1406274.

[67] Mahyuddin N, Awbi HB, Alshitawi M. The spatial distribution of carbon dioxide in rooms with particular application to classrooms. Indoor Built Environ. 2014;23(3):433–48. 10.1177/1420326x13512142.

[68] Lyu X, Luo Z, Shao L, Awbi H, Lo Piano S, Safe. CO(2) threshold limits for indoor long-range airborne transmission control of COVID-19. Build Environ. 2023;234:109967. 10.1016/j.buildenv.2022.109967. 36597420 10.1016/j.buildenv.2022.109967 PMC9801696

[69] Zhang S, Lin Z. Dilution-based evaluation of airborne infection risk - Thorough expansion of Wells-Riley model. Build Environ. 2021;194:107674. 10.1016/j.buildenv.2021.107674. 33583999 10.1016/j.buildenv.2021.107674 PMC7871780

[70] Fedele A, Colantoni A, Calabro G, Scungio M, Rossi S, Taborri J. Measuring CO(2) concentration and thermal comfort in Italian university classrooms: a seasonal analysis. Sens (Basel). 2025;25(7):1970. 10.3390/s25071970. 10.3390/s25071970 PMC11991519 40218481

[71] Borgese L, Tomasoni G, Marciano F, Zacco A, Bilo F, Stefana E, et al. Definition of an indoor air sampling strategy for SARS-CoV-2 detection and risk management: case study in kindergartens. Int J Environ Res Public Health. 2022;19(12):7406. 10.3390/ijerph19127406. 10.3390/ijerph19127406 PMC9224333 35742654

[72] Querol X, Alastuey A, Moreno N, Minguillon MC, Moreno T, Karanasiou A, et al. How can ventilation be improved on public transportation buses? Insights from CO(2) measurements. Environ Res. 2022;205:112451. 10.1016/j.envres.2021.112451. 34848209 10.1016/j.envres.2021.112451

