# Supplementary material for: Mitigating indoor risk of airborne infections using CO2 and PM measurements in university classroom: the MIRAI project
Source: J Environ Health Sci Eng. 2026 May 30;24(1):13. doi: 10.1007/s40201-026-00984-2 (PMC13222144; doi:10.1007/s40201-026-00984-2)

**Supplementary Figure S1.** Individual CO_2_ measurements in Unit 1 and Unit 2. Hourly aggregated values reported in ppm.


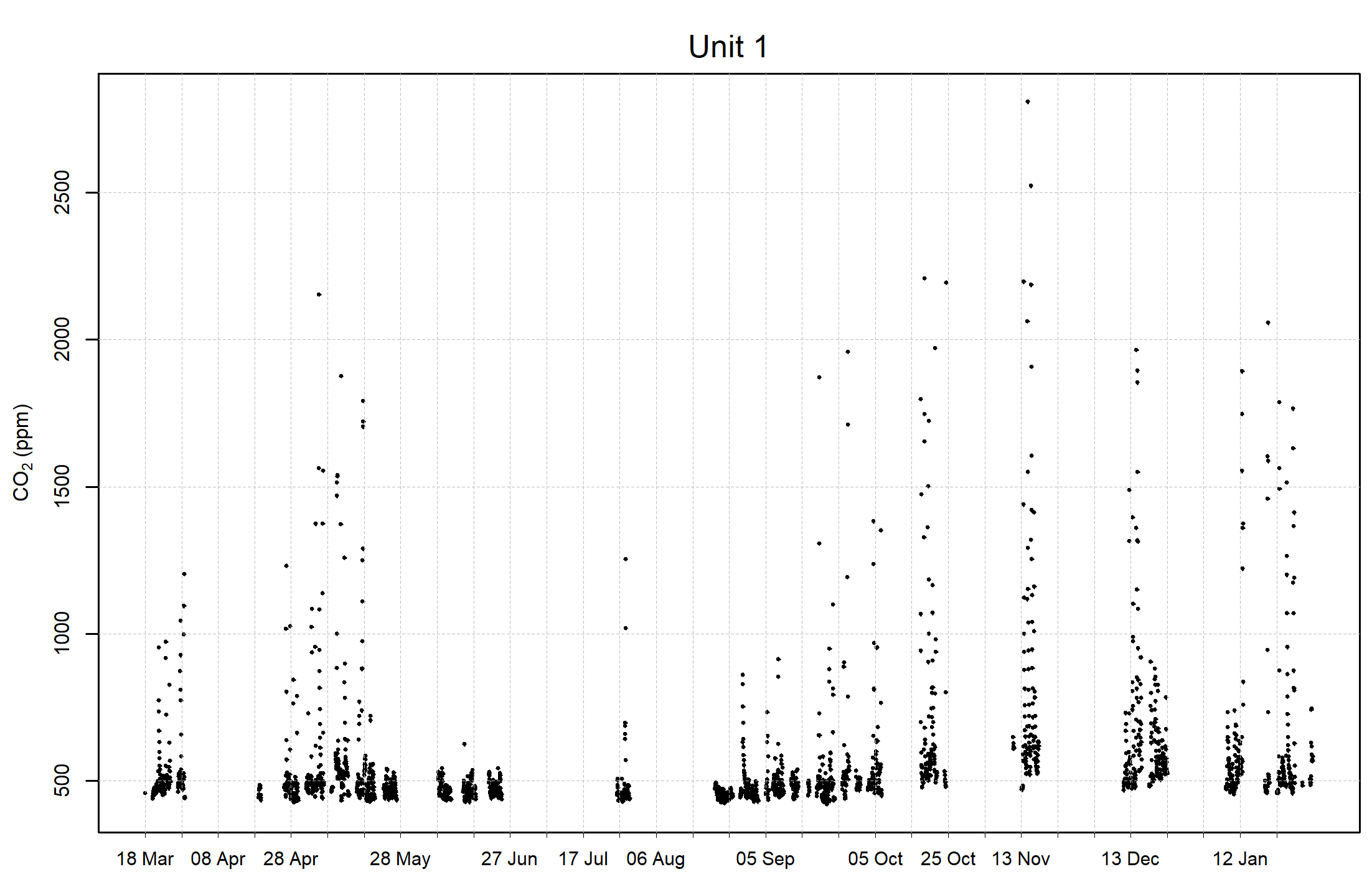


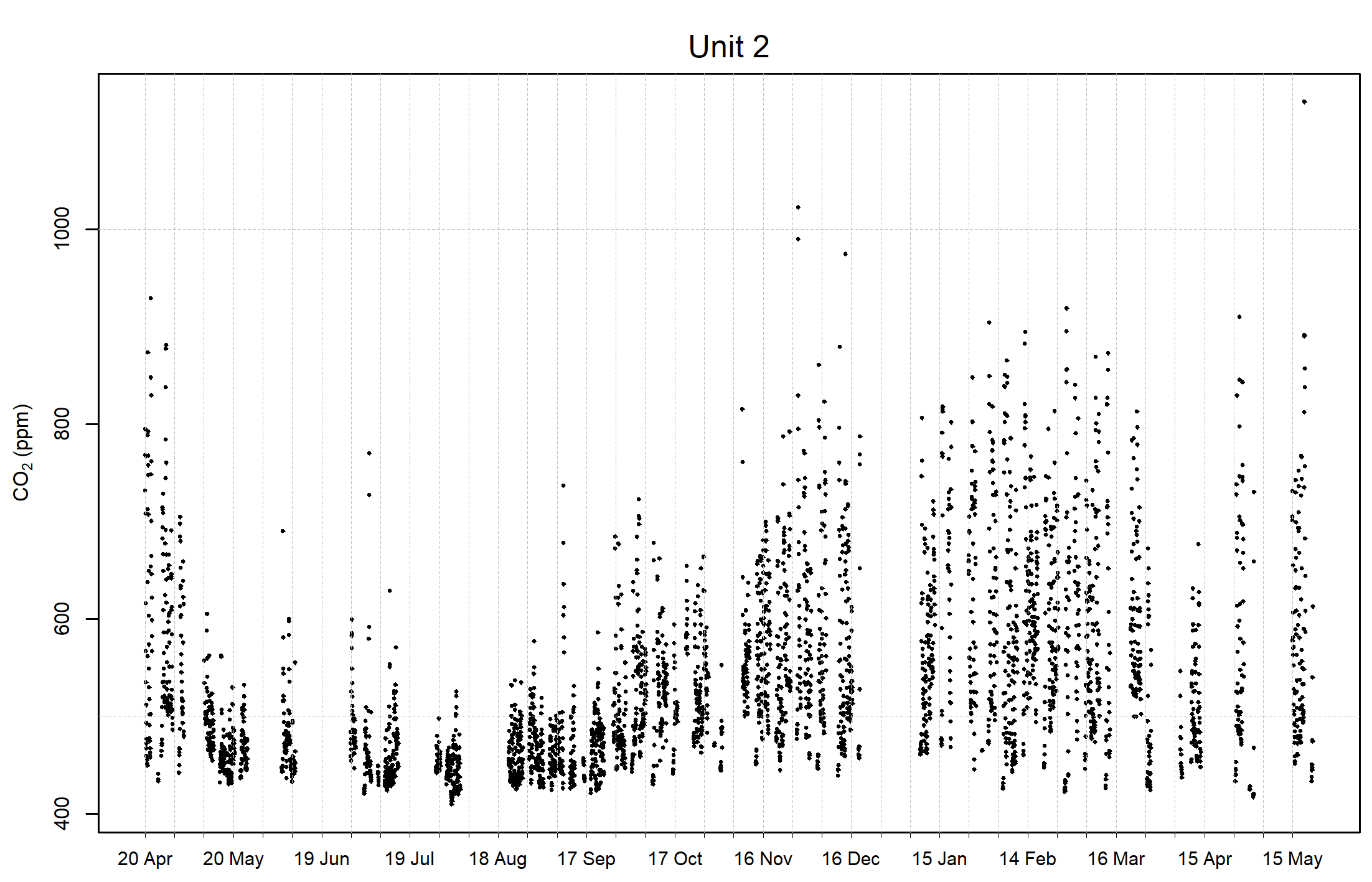


**Supplementary Figure S2**. Annual trends of total particle count and particle concentration. Hourly aggregated values during weekdays for Unit 1 (small, naturally ventilated classroom) and Unit 2 (large, HVAC-served classroom). Total particle count is expressed as number of particles per cm3; particle concentrations are expressed as µg/m_3_ (PM_1_, PM_2.5_, PM_10_) derived from the OPC-N3 size distribution.

Unit 1 (Public Health Department) Unit 2 (Engineering Department)


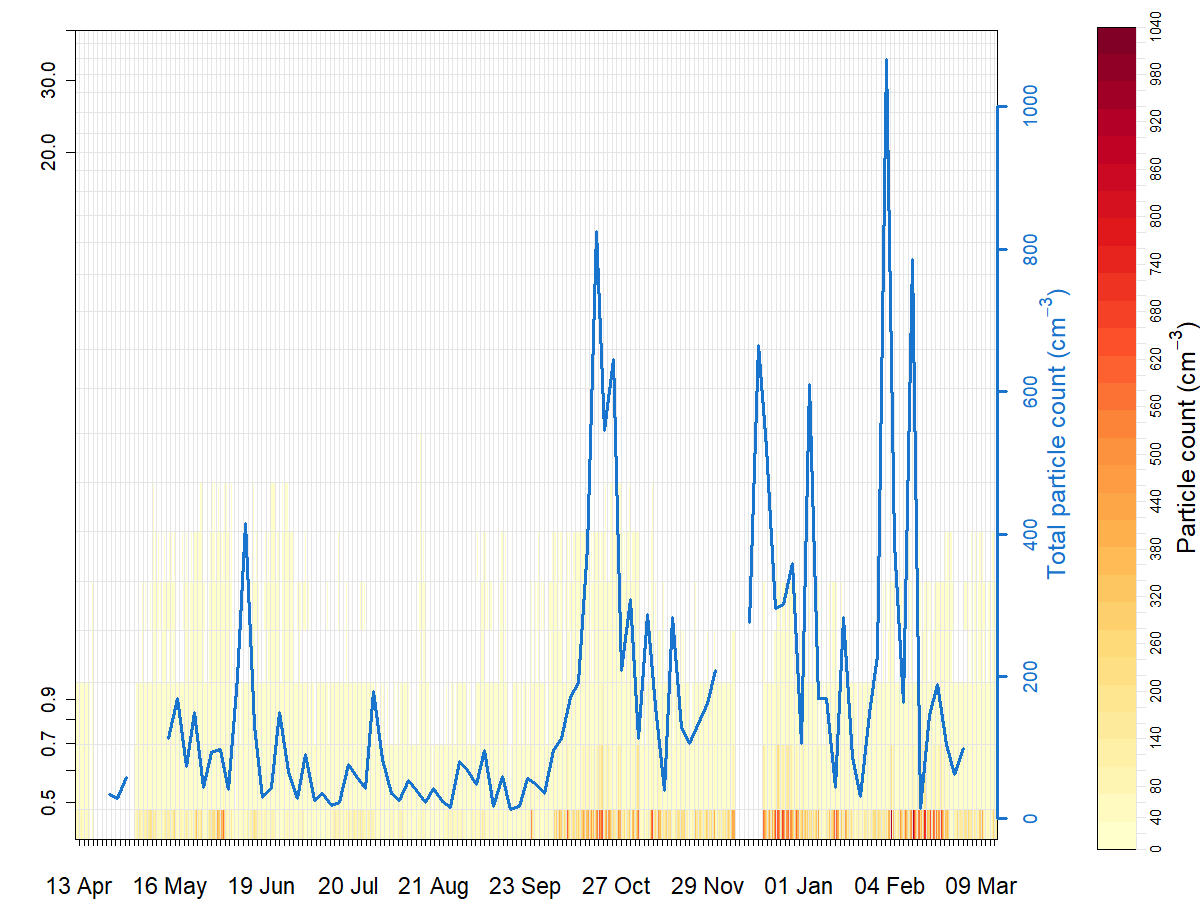

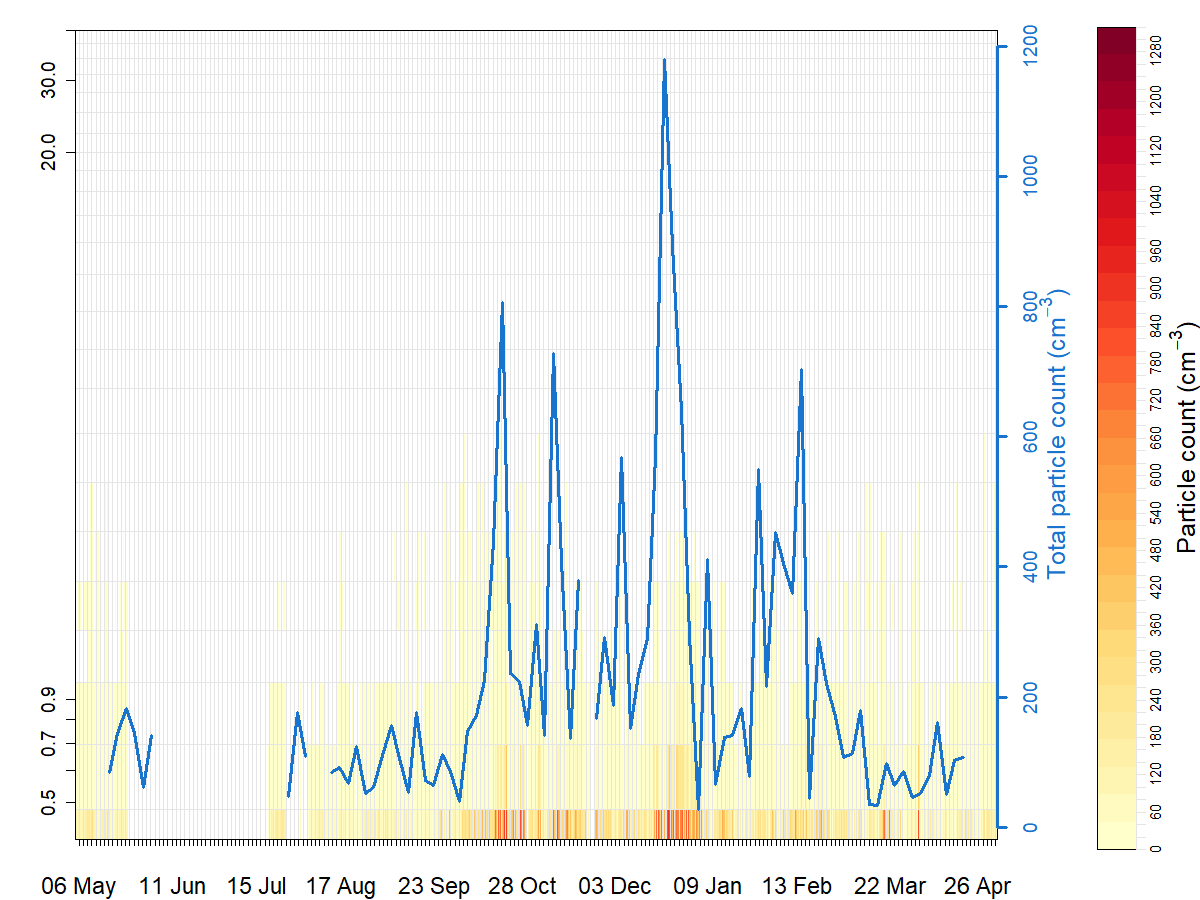

Supplement: Supplementary file 1 — Supplementary Material 1 [file 40201_2026_984_MOESM1_ESM.docx]
